# Carbon sequestration potential of natural vegetation under grazing influence in Southern Tigray, Ethiopia: implication for climate change mitigation

**DOI:** 10.1016/j.heliyon.2019.e02329

**Published:** 2019-08-30

**Authors:** Tesfay Atsbha, Anteneh Belayneh Desta, Tessema Zewdu

**Affiliations:** a Tigray Agricultural Research Institute, Alamata Agriculture Research Center, P.O. BOX 56, Alamata, Ethiopia; b School of Biological Sciences and Biotechnology, College of Natural and Computational Sciences, Haramaya University, Ethiopia; c College of Agriculture and Environmental Sciences, Haramaya University, Ethiopia

**Keywords:** Agricultural science, Environmental science, Ecology, Plant biology

## Abstract

The management influence on carbon sequestration potential of different land use types are least known at the national level. This research was conducted to assess the impact of area exclusion on carbon sequestration potential in the two land use systems: protected natural vegetation (PNV) and communal grazing land (CGL). Data of vegetation, litter, and soils were collected using systematic sampling methods, laying 19 transects and 62 quadrats each with 20 m × 20 m for trees, 5 m × 5 m sub-quadrats for shrubs, and 1 m × 1m sub-quadrats for herbs/grasses, litter biomass, and soil sample. Aboveground biomass carbon (AGC), belowground biomass carbon (BGC), soil organic carbon (SOC), and total carbon stock (TC) were estimated using allometric equations. The mean difference level of carbon stocks (P < 0.05) of the two land use systems was tested through unequal variance t-test using R-software. The mean above ground and below ground carbon stock of PNV, 21.05 ton/ha, 10.39 ton/ha, was higher than CGL, 15.31 ton/ha, 7.65 ton/ha, respectively. The average values of SOC was 16.60 ton/ha from PNV and 13.76 ton/ha from CGL. The mean value of SOC was higher at the PNV than CGL and significantly different (P < 0.05). The total carbon stock estimate of PNV and CGL were 50.74 ton/ha and 37.11 ton/ha, respectively, which is significantly different (P < 0.05). We concluded that, establishment of PNV as the best practice of restoration programs through exclusion of livestock from free grazing and human interference provides cost effective mechanism that yields a high carbon sequestration potential with multiple benefits for biodiversity conservation, livelihood support, and climate change mitigation.

## 1 Introduction

The decline of vegetation cover is one of the most serious environmental issues faced humankind today. In this respect, Ethiopia is facing severe degradation in both the highlands and the lowlands. Particularly, the Ethiopian grazing land ecosystems were faced challenges of intense degradation due to deforestation, agricultural land expansion, and continuous and heavy grazing (Lemenih et al., 2005; Mengistu et al., 2005a; Gebrewahid et al., 2018). Overgrazing is one of the major factors aggravated ecosystem degradation, which causes an increase in unpalatable species by destroying the most palatable species and reduce plant cover and biomass, thereby increase erosion hazard and reduce the overall productivity of the land (O'Connor et al., 2001; Bot and Benites, 2005; Pulido et al., 2018). The country was striving for different conservation strategies in order to minimize such threats like, watershed management, afforestation, and reforestation, restoration, and rehabilitation programs (Mengistu et al., 2005b). Such practices were found significantly improve the vegetation cover and contributes to the livelihoods of local communities (Mebrat, 2015; Wolde et al., 2018) and used as strategies for climate change mitigation (Bikila et al., 2016; Chiemela et al., 2018). As part of this national initiative the country has been broken the global record by planting more that 350 million tree seedlings per day on 29July 2019. Especially, the northern part of Ethiopia known as Tigray Regional State was much effective in practicing diverse conservation strategies on the severely affected and degraded landscapes for decades, in which area exclusion of livestock from free grazing and human interference were the most common (Solomon et al., 2017; Atsbha et al., 2019). In this part of the country, establishment of area as protected natural vegetation (PNV) has been taken as a management strategy for the rehabilitation or restoration of degraded hillsides (Tesfay, 2011).

Afforestation (tree planting) and rangeland restoration through grazing exclusion are the two widely suggested options for ecosystem improvement and carbon sequestration (Gebrehiwet and Van der Veen, 2014; Atsbha et al., 2019). Forestations can increase carbon influx through a higher and more efficient use of resources for primary production (Belayneh et al., 2011). Grazing exclusion is likely to increase carbon uptake where overgrazing has reduced plant cover and/or impaired soil fertility (O'Connor et al., 2001; Seyoum et al., 2015). In addition to carbon uptake shifts, afforestation and grazing exclusion can favors carbon sequestration through the reduction of carbon losses if higher ground cover reduces soil organic carbon (SOC) decomposition and soil erosion (Jackson et al., 2002; Wolde et al., 2018).

Rehabilitation of degraded communal grazing lands through establishing PNV has become increasingly important in Tigray region, northern Ethiopia. Hence, approximately 1.5 million hectares of land has been excluded from grazing in the last three decades in the region (Seyoum et al., 2015). However, information on carbon sequestration potentials of degraded grazing lands in the Tigray region of Ethiopia following the management practices such as area exclusion is lacking under the ever-changing climate. Such management influence on carbon sequestration potential of different land use types are least known in the country. Hence, this study was designed to determine carbon sequestration potentials of the two land use types, protected natural vegetation (PNV) and communal grazing land (CGL), of Gra-Kahssu natural vegetation. This would help to evaluate the impacts of conservation and management practices to support the planning process in effective conservation strategy for multiple benefits i.e. biodiversity conservation, livelihood support, and climate change mitigation.

## 2 Materials and methods

### 2.1 Description of study area

**Fig. 1:**
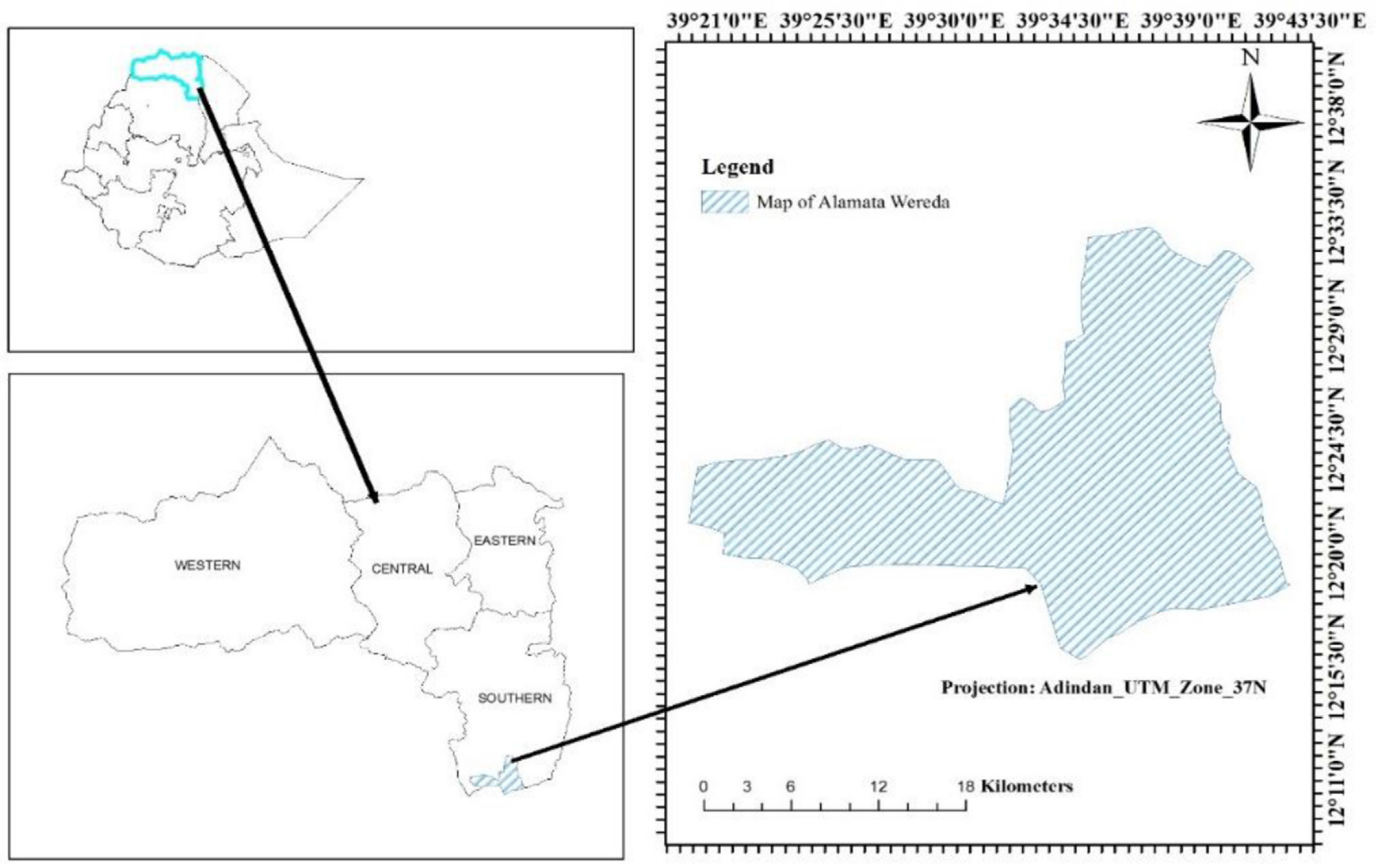
Location map of the study.

The study area, Gra-Kahsu national forest priority area in Alamata Wereda (district) is located 600 km north of Addis Ababa and 180 km south of Tigray regional state capital (Mekelle). It is geographically located between 12°19′21″N and 12°24′28.5″ North latitude and 39°14′52″E and 39°45′47.8″ East longitude in Southern Tigray (Fig. 1). Alamata is borders with Amhara region from the south and west and Afar region from the East. The altitude of the Wereda ranges from 1,178 to 2,300 meters above sea level (m.a.s.l). About 75% of the Wereda can be described as lowland (1,500 m.a.s.l or less), and the remaining 25% is in the mid-highlands (range between 1,500–2,300 m.a.s.l). The altitudinal of the study area, Gra-Kahssu natural vegetation, ranges from 1620 - 2298 m.a.s.l. The annual mean precipitation ranges from 615-900 mm, with maximum and minimum temperatures of 23 °C and 14 °C, respectively (Girmay et al., 2014). In the study area, Gra-Kahsu national forest priority area, endemic, indigenous, and multipurpose plant species like, *Acacia abyssinica, Celtis africana, Clutia abyssinica, Cynanchum abyssinicum, Dovyalis abyssinica, Hagenia abyssinica, Maytenus undata, Myrsine africana, Osyris quadripartite, Phytolacca dodecandra, Pittosporum viridiflorum, Rosa abyssinica, Rhus glutinosa* and *Teclea simplicifolia* were found increasing in the PNV. Whereas, woody species like *Euclea racemosa, Acacia tortilis, Gomphocarpus fruticosus*, and *Carissa spinarum* were increasing dominance in the CGL (Atsbha et al., 2019). The major fauna found at Gra-Kahsu national forest priority area are Monkeys, Ethiopian Tiger (*Panthera tigris*), Menelik Bushbuck (*Tragelapphus scriptus*), Python (Snake type), Fox and diverse species of birds (WAOARD, 2016).

### 2.2 Sampling method

Reconnaissance survey was made throughout the study areas prior to the field layout for vegetation, biomass, and soil sampling. Accordingly, sampling sites were identified in the protected natural vegetation (PNV) of 3500 ha and communal grazing land (CGL) of 3700 ha for data collection. Each land use types were further stratified based on on homogeneity in floristic composition and distributional pattern. Then systematic transect sampling technique was used to collect the vegetation and soil data within the strata of the two land use systems (PNV and CGL). A parallel line transects were laid at 500m interval that lie with parallel to the slope of the stand (19 transect sampling, 10 from PNV and 9 from CGL). The first quadrat was laid randomly and the others systematically at 500m intervals along the transect line, which were laid parallel to the slope. The locations of the quadrats were marked by GPS and slope along transects were measured using clinometers. Along the transect lines, a total of 62 quadrats (31 from PNV and 31 from CGL) measuring 20 m × 20 m for trees, 5 m × 5 m sub-quadrats for shrubs, and 1 m × 1 m sub-quadrats for herbs/grasses, litter, and soil were laid down (Gemedo et al., 2006).

### 2.3 Data collection methods

#### 2.3.1 Woody species

The data collection on woody vegetation, herbs, grasses, litter, and soil samples were carried out between September and December 2017. Trees measuring ≥2.5 cm in diameter at breast height (DBH), approximately 1.30 cm above the ground, and shrub diameter at stump height (DST), about 30 cm from the ground, were measured from each quadrat of the corresponding size. In case, if the stem of a tree was branched at breast height or below, the diameter of separate branches were measured to consider as individual tree similarly, applied to multi-stemmed shrubs (Snowdon et al., 2002). In addition, if a given tree or shrub stems are buttressed, its DBH or DSH was measured 5 cm above the buttresses point. A calibrated bamboo stick having 5m height graduated with 10 cm markings was used for measuring the height of longer tree species whereas a Sunto clinometer was used for trees greater than 5 m in height. The biomass and carbon stock of dominant trees and shrubs were estimated using allometric equations developed for tree and shrub species (Henry et al., 2011). Then, biomass-carbon conversion factor of 0.5 was used to calculate the above-ground biomass carbon of woody species (Liu et al., 2014). In addition, the below-ground biomass for trees and shrubs was estimated from root-shoot ratios by taking into account the 27% of above-ground biomass of woody species (Penman et al., 2003).

#### 2.3.2 Herbaceous and litter biomass

Destructive sampling method was used for measuring the biomass of grasses and herbs by harvesting whole parts of fresh samples within each quadrat, a size of 1 m × 1 m, using sickle from. All the herbaceous vegetation emerging within the quadrat areas were cut at the ground level, weighed, and a composite sample was obtained from each sub-quadrat for oven-dry mass determination in the laboratory (Dossa et al., 2008; Jina et al., 2008). Similarly, surface litter was sampled from the sub-quadrats (1 m × 1 m) and composite litter was collected. The herb and litter samples (62 herb and 62 litters) were weighed using a digital scale (0.1-gram accuracy) and recorded. The samples were mixed well and 10–20% sub-sample was taken for dry to fresh biomass ratio. The samples collected were subjected to air and oven drying. Oven drying was set at 70 °C and observed for 24 hours or until the samples reached their stable weight (Labata et al., 2012). Herbaceous vegetation carbon stocks were calculated as 50% of oven-dried herbaceous biomass (Pearson et al., 2005).

#### 2.3.3 Soil organic carbon stock

The soil samples were collected in each quadrat along the transect line at three levels of soil depths i.e. 0–10, 10–20 and 20–30 cm using a soil auger. Then one soil core sample was taken from the centre of each transect line for soil bulk density (SBD) determination (Subedi et al., 2010; Gebrewahid et al., 2018). An equal weights of each sample of the corresponding depth were pooled and mixed together from a given transect line, air dried and passed through a 2 mm sieve to separate debris and gravel. Finally, composite samples were equally divided into four and one has been randomly taken as a representative sample, which has been packed in plastic bags, labelled, sealed and transported to the soil laboratory. A total number of 57 soil samples were taken for bulk density determination. Thirty soil samples (10 transect x 3 soil depth) from PNV and 27 soil samples (9 transect x 3 soil depth) from CGL were collected. Then the soil sample was air-dried followed with oven dried at 105 °C for 24 hours at Mekelle soil laboratory research centre. Bulk density was measured using the core method (Pearson et al., 2005) and soil organic carbon was determined by Walkley–black method (Black et al., 1965).

## 3 Analysis

### 3.1 Above ground biomass (AGB)

The AGB of trees ≥2.5 cm in DBH and ≥1.5 m in height estimated using the allometric model of Kuyah et al. (2012). The equation is as follows:

(1) AGB = 0.1428∗DBH^2.2471^

AGB diameter measures at DSH of multi-stem trees and shrubs were estimated from DSH using the equation is as follows:

(2) AGB=(0.4861∗DSH)+(0.1659∗(DSH^2.2^)

To convert the above ground dry biomass to carbon, 50% of all trees and shrubs biomass were assumed the carbon stock. So based on the aboveground trees and shrubs biomass carbon stock calculated as follows (Brown et al., 2002):

(3) AG TSCS = AG TSDBM ∗ 0.5

where; AGTSCS: Above ground trees and shrubs carbon stocks; AG TSDBM: Above ground trees and shrubs dry biomass.

### 3.2 Below-ground biomass (BGB)

To measure BGB, It was used root-to-shoot ratio, which has become the standard method for estimating root biomass from the more easily measured shoot biomass. The equation developed by MacDicken (1997) to estimate belowground biomass was used. The equation is given below:

(4) BGB = AGB × 0.2 (2)

Where, BGB is below ground biomass, AGB is above ground biomass, 0.2 is conversion factor (or 20% of AGB).

### 3.3 Carbon stocks in the herb and litter layer biomass

Oven-dry weights of herb and litter subsamples were determined to compute for the total dry weights using the formula (Hairiah et al., 2001):

(5) Total dry weight (kg m^−2^) = Total fresh weight (kg) * subsample dry weight (g)/ Subsample fresh weight (g) * Sample area (m^2^)

Carbon storage in herb and litter layer was computed using the formula (Lasco et al., 2006):

(6) C stored (ton/ha) = Total dry weight ∗ C content

The carbon stock (carbon content) for the dry biomass of herbs and litters is 47% of the total dry biomass of the quadrate (IPCC, 2007).

### 3.4 Dry biomass and carbon stock in the dead wood

Dead wood biomass was computed using the formula (Pearson et al., 2005):

(7) BSDW = 0.139DBH^2.32^–5.5%

Where, BSDW = Biomass of standing dead wood in ton/ha, DBH = Diameter at breast height of standing dead wood (cm)

The total carbon stock in dead wood was computed by multiplying the total biomass of the dead wood by 0.5 (Pearson et al., 2005).

### 3.5 Soil organic carbon (SOC)

Soil bulk density was determined after oven drying the soil samples that are taken with core sampler as follows formula as recommended by Pearson et al. (2005).

(8) V = h∗πr^2^

Where: - V = volume of the soil in the core sampler in cm^3^, h = the height of core sampler in cm, π = 3.14 cm, r = the radius of core sampler in cm. Moreover, the bulk density (ρb) of a soil sample was calculated as follows:

(9) ρb = Wav,dry/v

Where, ρb is bulk density of the soil sample per quadrate (g cm^−3^), Wav, dry is average air dry weight of soil sample per the quadrate, V is volume of the soil sample in the core sampler auger in cm3 (Pearson et al., 2005).

Collected composite soil samples were examined for SOC estimation using the Walkely-Black methods (Black et al., 1965). SOC per quadrate and then per hectare in tons calculated as follows:

(10) SOC = (ρb (g cm^3^) ∗ D (cm) ∗ %C)

Where, SOC = Soil organic carbon (t/ha), % OC = Organic carbon concentration of the quadrate (%) expressed in decimal, ρb = Bulk density of the quadrate (g cm^−3^), D = Depth of the soil sample (cm).

### 3.6 Total carbon stock estimate

The total carbon stock from various carbon pools was calculated by aggregating the carbon stock densities of the individual carbon pools using the equation given by Subedi et al. (2010). The total carbon stock is then converted to tons of CO_2_ equivalent by multiplying it by 44/12, or 3.67 (Pearson et al., 2007).

(11) TC = AGC + BGC + DWCS + SOC

Where TC is total carbon, AGC is aboveground carbon, BGC is belowground carbon, DWCS is dead woody carbon stock, and SOC is soil organic carbon.

T-test unequal variance using R-software was employed to test the level of significance difference of carbon stock of the two land use systems. Analysis of variance (ANOVA) also used to analysis the mean of soil organic carbon across the soil depth. The least significant difference (LSD) was used to separate the means. Differences were considered significant at P < 0.05.

## 4 Results and discussion

### 4.1 Estimation of above and belowground carbon stocks

The above ground carbon stock of protected natural vegetation (PNV) was ranged between 11.59 and 42. 22 ton/ha with the mean value of 21.05 ton/ha whereas, the adjacent communal grazing land (CGL) ranged between 7.16 and 32.04 ton/ha with the mean value of 15.31 ton/ha (Table 1). The above ground carbon stock was higher in the PNV compared to the CGL. The algometric estimation of below ground carbon stock, with 0.5 times that of the above ground carbon, sill indicated higher in PNV compared to CGL (Table 1). The potential drivers like over grazing and human interference for firewood collection, tree/shrub cutting for fencing and construction, and charcoal making could be attributed to lower the carbon stocks in communal grazing land. Free grazing in the communal grazing land aggravated soil and vegetation degradation, which in turn negatively affected vegetation restoration and accumulation of aboveground biomass (Wolde et al., 2018) where the carbon stock is calculated. In addition, the management practices, basal area, species richness, the number of individuals per ha, species diversity and species composition were the probable reason for the differences in the AGC and BGC among the two-land use systems. For instance, Raju (2012) reported that, basal area is an important parameter, which determines the carbon content by the species. The more basal area and diameter indicated the more biomass (Solomon et al., 2017) resulted in the more carbon storage. Dead woody plant species recorded from nine quadrats in PNV and none in the CGL indicated the frequent utilization of dry woods by the local community.

**Table 1:** Carbon stocks (ton/ha) per hectare (mean ± SD) under the two land use systems.

Variables	Land use system						P value
	Protecting natural vegetation			Communal grazing land			
	Maximum	Minimum	Mean ± SD	Maximum	Minimum	Mean ± SD	
AGCS	42.21	11.58	21.05 (±7.56)	32.04	7.16	15.32 (±6.18)	<0.001
BGCS	21.10	5.79	10.40 (±3.68)	16.02	3.58	7.66 (±3.09)	0.001
DWCS	6.65	0.76	2 (±1.86)	-	-	-	

AGC = Aboveground woody carbon stocks; BGCS = Belowground carbon stocks; DWCS = dead woody carbon stocks; SD = Standard deviation.

### 4.2 Herbs and litter biomass carbon stocks

The biomass carbon stock of herbs and litters were showed statistically significant difference (P < 0.001) between the two-land use systems (Table 2). The litter carbon stock was significantly lower in the CGL compared to the PNV. The mean carbon stock of herb and litter biomass in PNV was 0.60 and 0.10 ton/ha, and 0.34 and 0.03 ton/ha in adjacent CGL, respectively. The reason for relatively higher litter and herb biomass carbon stock in PNV could be due to the presence of higher species density, and less human and livestock interference than in CGL. A higher herbs and litters carbon stocks in the PNV compared to the adjacent communal grazing land in our study was consistent with the report of Solomon et al. (2017) where the herbaceous biomass were significantly higher in exclosure than the adjacent communal grazing land. This might be due to the fact that continuous heavy grazing in communal grazing land inhibits the growth of herbaceous layers and decrease aboveground herbaceous biomass through direct removal, leading to the depletion of herb and litter carbon stocks. Similar reports indicated a significant difference in herbaceous carbon stocks between PNV and the adjacent communal grazing land (Mekuria, 2013; Bikila et al., 2016). The significant increase of herb and litter carbon biomass compared to the communal grazing land may be indicates the potential for the restoration of the herb and litter biomass through range restoration.

**Table 2:** Herb and litter mean (±SD) biomass carbon stock of both land use systems (ton/ha).

Variables	Land use system						P value
	Protecting natural vegetation			Communal grazing land			
	Maximum	Minimum	Mean ± SD	Maximum	Minimum	Mean + SD	
HCS	1.42	0.08	0.59 ± 0.30	1.03	0.09	0.34 ± 0.18	<0.001
LCS	0.29	0.02	0.10 ± 0.67	0.11	0.01	0.03 ± 0.02	<0.001

HCS = herbs carbon stocks; LCS = litter carbon stocks; SD = Standard deviation.

### 4.3 Soil organic carbon

The soil organic carbon (SOC) showed variation among the different soil depth in both land use systems. Statistically significant differences (P < 0.05) were observed in soil organic carbon across the three soil depth profiles i.e. 0–10 cm, 10–20 cm and 20–30 cm (Table 3), which has been reduced down across the soil depths. The pattern indicates that soil carbon was highly accumulated in the upper soil layers at both land use systems. Similar reports by Abule et al. (2005); Fantaw et al. (2006); Sheikh et al. (2009); Fynn et al. (2009); Swai et al. (2014); Sheikh et al. (2014) and Bikila et al. (2016) indicated that soil organic carbon has been reduced down across the soil depth. This may be due to the accumulation and rapid decomposition of natural vegetation litter in the top soil. Mendoza-Vega et al. (2003); Chowdhury et al. (2007); Ullah and Al-Amin (2012) and Muluken et al. (2015) found that more SOC was stocked at the soil depth of 0–14 cm. It has been also reported that the mean SOC stock was significantly higher at 0–10 cm depth and lower at 30–40 cm soil depth (Koul et al., 2011). Soil Organic Carbon concentration in the three different depths differed significantly (P < 0.001) among the two land use systems.

**Table 3:** Means (±SD) soil organic carbon across the soil depth (cm) at both land use systems (ton/ha).

SOC of land use types	Different depths		
	0–10 cm	10–20 cm	20–30 cm
Protecting natural vegetation	20.08 ± 5.06^a^	15.67 ± 3.07^b^	14.05 ± 2.81^b^
Communal grazing land	16.83 ± 6.09^a^	12.69 ± 2.91^ab^	11.74 ± 3.41^b^

Different letters in the same column are significantly different (P < 0.05).

The mean values of soil organic carbon were 16.60 ± 4.45 and 13.76 ± 4.76 ton/ha for PNV and CGL, respectively. This value indicated that, the mean value of SOC was higher at the PNV than CGL and have significantly difference (P < 0.05) (Table 4). It is consistent with the finding of Derner and Schuman (2007); Wolde and Aynekulu (2011); Bikila et al. (2016), who reported that restoration of rangelands soils and ecosystems with permanent vegetation has high potential for soil carbon sequestration. The differences in SOC stocks between the PNV and CGL could be attributed to an increased vegetation biomass and the subsequent production and decomposition of litter fall from the vegetation that could add more organic matter into the soil systems. Thus, grazing lands with more aboveground vegetation biomass contribute more to carbon sequestration potential of the soil as compared to CGL having less aboveground vegetation biomass. This result agreed with the work of Bikila et al. (2016) and Mekuria (2013) who reported that SOC was higher for PNV than open grazing areas. Sheikh et al. (2009) also reported that high SOC mass was recorded in the protected areas compared to open grazing lands. The management practice through area exclosure has been contributed to increased soil organic carbon. Exclosure, which removed grazing pressure and allowed regeneration of native vegetation, increased SOC in the top soil (Assefa et al., 2017). Natural forest always had highest SOC content due to the presence of vegetation that enhances the SOC content through its continued production and decomposition of litter biomass (Koul et al., 2011). The mechanism of species driven carbon sequestration in soil was influenced by two major activities, aboveground litter decomposition and belowground root activity (Lemma et al., 2007).

**Table 4:** Means (±SD) of soil organic carbon across land use system (ton/ha).

SOC of land use types	Maximum	Minimum	Mean ± SD	P value
Protecting natural vegetation	28.41	7.20	16.60 ± 4.47	0.04*
Communal grazing land	30.52	5.72	13.76 ± 4.76	

SOC = soil organic carbon; SD = Standard deviation; *Significant difference level.

### 4.4 Total carbon stock

The total carbon stock in the study area was 50.74 tons/ha and 37.11ton/ha for PNV and CGL, respectively (Table 5). With regard to total carbon in PNV, relatively higher carbon amount was observed in all the six carbon pools compared to the value found in all carbon pools of the CGL. This is attributed to the presence of higher species richness, diversity, density, DBH, litter and herb biomass compared to the CGL, which makes the PNV higher in total carbon stock. Significant differences between the exclosure and adjacent grazing land in woody species richness, diversity and evenness was reported (Wolde et al., 2018), which has been contributed for higher total carbon stock in PNV compared to the adjacent CGL.

**Table 5:** Means (±SD) of total carbon across land use system (ton/ha).

TCS of land use types	Maximum	Minimum	Mean ± SD	P value
Protecting natural vegetation	100.08	25.43	50.74 + 17.91	<0.001**
Communal grazing land	79.72	16.56	37.11 ± 14.21	

TCS = total carbon stock; SD = Standard deviation; **Significant difference level.

**Fig. 2:**
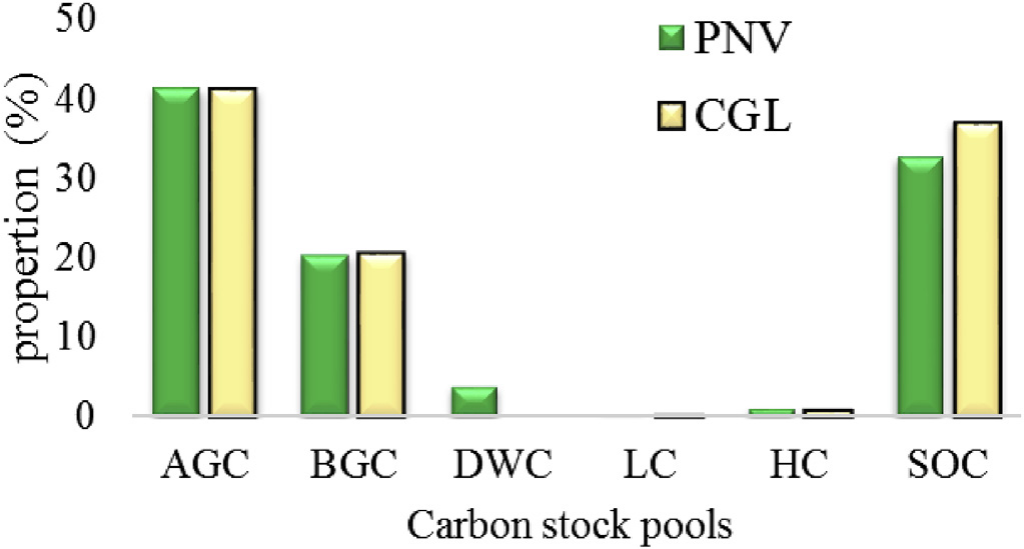
The proportion of each carbon pools from the total carbon stock of both land use systems. (AGC - Above ground carbon; BGC - Below ground carbon; HC - Herb carbon; LC - Litter carbon; DWC-Dead wood carbon; SOC - Soil organic carbon).

In both land use systems, the proportion of carbon stock was higher for above ground carbon, which is 41.49% and 41.28% in PNV and CGL, respectively. The second highest carbon stock proportion was the soil organic carbon and its percentage value 32.72% and 37.08% in the PNV and CGL, correspondingly (Fig. 2). Unlike this report, Oliva-García et al. (2006) reported that 51% by proportion from the total carbon stock of tropical forest of Mexico was found in the soil. Normally, soils are the largest carbon pools in global terrestrial ecosystems, because they can contain three times more carbon than that contained in vegetation (Gelaw et al., 2014).

The result was consistent with Muluken et al. (2015) who reported that, the above ground biomass was the highest carbon stock followed by soil organic carbon at the Gambella National Park and Adaba-Dodola community forest of Danaba District, West-Arsi zone of Oromia Region Ethiopia, respectively. Abel et al. (2014) at the Mount Zequalla Monastery, Eastern Shewa Zone and Worku (2016) at the Gendo moist Montane forest, East Wollega Zone, Oromia National, Regional State also reported that, the proportion of carbon pools was AGC > SOC > BGC > HC as decreasing order.

## 5 Conclusion

The mean carbon stock in all carbon pools of the PNV was found greater than that of the CGL. The carbon dioxide equivalent (CO_2_e), which indicates the carbon dioxide emitted or sequestered in the PNV was 186.22ton/ha where as the carbon sequestration of the CGL was 136.19 tons/ha CO_2_e. The management influence like area exclusion, which has been practiced in PNV was contributed to increased species richness, diversity, density, litter and herb biomass, and soil organic carbon compared to the CGL, which makes PNV higher in total carbon stock. Because, the carbon losses from the different land use types are associated with loss of vegetation cover and soil erosion (Sheikh et al., 2014; Wolde et al., 2018). Therefore, we concluded that establishment of exclosures, with least interference of human and livestock, on degraded lands could support the restoration of degraded native vegetation and soil properties, which consequently enhance the carbon sequestration potential of the ecosystem as well as support the ongoing climate change mitigation strategy in the country in general and Northern Ethiopia, Tigray Region, in particular. Therefore, opportunities for improved grazing land management as well as increasing carbon sequestration should be developed and enhanced in degraded land condition, which will also have significant contribution to the household economy by providing opportunities to diversify livelihood. Finally, we recommended, the need for further studies on temporal and spatial vegetation biomass and carbon stocks should be thoroughly investigated to capture the whole dynamics of the grazing land ecosystems under various regimes of grazing exclusions.

## Declarations

### Author contribution statement

T. Atsbha: Conceived and designed the experiments; Performed the experiments; Analyzed and interpreted the data; Contributed reagents, materials, analysis tools or data; Wrote the paper.

A. Belayneh Desta: Conceived and designed the experiments; Analyzed and interpreted the data; Wrote the paper.

T. Zewdu Kelkay: Analyzed and interpreted the data; Wrote the paper.

### Funding statement

This work was supported by Tigray Agricultural Research Institute through Alamata Agricultural Research Centre.

### Competing interest statement

The authors declare no conflict of interest.

### Additional information

No additional information is available for this paper.
